# Synthesizing five decades of research on sensitive caregiving: A commentary on Nivison et al. (2026)

**DOI:** 10.1111/jcpp.70146

**Published:** 2026-03-18

**Authors:** K. Lee Raby

**Affiliations:** ^1^ Department of Psychology The University of Utah Salt Lake City UT USA

## Abstract

This commentary highlights the contributions of Nivison et al.'s (2026) umbrella meta‐analysis synthesizing five decades of research on sensitive caregiving and child development. Integrating findings from numerous meta‐analyses, the authors demonstrate that caregiver sensitivity is meaningfully associated with multiple domains of child development. Notably, associations with cognitive and language development are at least as large as those with attachment security and behavior problems, expanding traditional conceptualizations of sensitivity's developmental significance. The findings further indicate substantial consistency across child, parent, and family demographic characteristics, while suggesting amplified benefits in socioeconomically disadvantaged contexts. This commentary underscores key gaps in the literature, including the need for meta‐analytic investigations of children's peer competence, self‐regulation, and physical health outcomes, as well as the need for refined measurement of caregiving dimensions. Although causal inferences require randomized intervention evidence, the synthesis provides compelling support for sensitive caregiving as a central determinant of healthy development and offers a roadmap for future research and policy.

The paper by Nivison and colleagues quantitatively reviews a vast body of research on the developmental consequences of sensitive caregiving that stretches back over five decades. During Mary Ainsworth's (1967) field study of mothers and infants in Uganda, she made a groundbreaking observation: individual differences in infants' attachment behaviors were associated with aspects of their mother–infant interactions. Later, Ainsworth, Blehar, Waters, and Wall (1978) built on these initial observations by (a) defining sensitive caregiving as awareness and accurate perceptions of infants' signals as well as timely and appropriate responses to those signals, (b) creating a scale for rating sensitive caregiving during caregiver–infant interactions, and (c) conducting a longitudinal study that demonstrated that mothers' sensitive caregiving during the first year of life was a strong predictor of whether their infants eventually formed secure attachments to them.

These research methods and findings inspired a substantial number of studies examining the consequences of caregiver sensitivity for child attachment security under different conditions. These studies included caregivers other than mothers, children at older ages, and families from diverse socioeconomic backgrounds. At the same time, researchers widened the scope of this body of research by examining other child development outcomes, including children's cognitive, linguistic, and socioemotional functioning. Over the years, this expansive body of research has been summarized in nearly a dozen meta‐analyses. Many of these meta‐analyses focused on a specific child development outcome or a specific type of caregiver. As a result, the findings from these meta‐analyses have remained fragmented.

## Contributions of Nivison et al. (2026)

The paper by Nivison et al. (2026) integrates these disconnected literatures by providing a comprehensive synthesis of the available meta‐analyses on the child development outcomes associated with sensitive caregiving. This umbrella meta‐analysis serves two valuable functions. First, it allows for comparing the strength of the associations between sensitive caregiving and different domains of child functioning with a high degree of statistical power. Second, it allows for an evaluation of the consistency of moderation effects. In the same way individual meta‐analyses allow for testing whether a putative moderator replicates across studies, an umbrella meta‐analysis allows for testing whether a moderator replicates across developmental outcomes.

The results of Nivison et al.'s (2026) analyses demonstrate that sensitive caregiving is significantly associated with each of the four broad domains of child functioning that were examined. Most of the effect sizes are medium in overall magnitude by contemporary standards. In fact, the strength of the associations with children's cognitive and language skills is at least as large as those for children's socioemotional outcomes such as attachment security and behavior problems. This finding is especially novel and important because attachment researchers have traditionally emphasized the role of caregiving experiences in shaping children's mental health trajectories rather than their cognitive or linguistic skills (e.g., Sroufe, 1988). Across all the child development outcomes, the effect sizes are similar for cross‐sectional and longitudinal designs, which suggests that the effects do not fade over time (Fraley & Roisman, 2015). Putting all this together, Nivison et al. (2026) indicate that the associations between caregiver sensitivity and child development are *broad* in their scope, *meaningful* in their size, and *persistent* over time.

Nivison et al.'s (2026) analyses regarding potential moderators also reveal that the associations between sensitivity and child outcomes are highly consistent across key demographic characteristics of children, parents, and families. For example, the strength of the associations between sensitive caregiving and children's outcomes is similar for mothers and fathers. Although research in this area has historically focused on mothers, these findings indicate that sensitive caregiving has similar consequences regardless of the biological sex of the parent. Other research suggests that experiences with mothers and fathers have nonoverlapping contributions to children's development (Dagan et al., 2024). Thus, the combined effects of children's experiences of sensitive caregiving across all caregivers likely are larger than the estimates based on a single dyadic relationship. Nivison et al. (2026) also reported that associations between sensitivity and children's outcomes were quite similar regardless of the socioeconomic backgrounds of the families. In other words, although sensitive caregiving may be more prevalent among parents with more formal education and financial resources, the variation in caregiver sensitivity that nonetheless exists within each socioeconomic stratum has similar associations with children's development. In the single instance in which the effects varied across family socioeconomic status, the results demonstrated that the implications of sensitivity for children's language development are largest among families experiencing the most socioeconomic hardship. These findings have profound importance for social policy because they suggest that families at heightened risk for providing insensitive care will benefit from programs that promote this type of parenting at least as much as more advantaged families.

## Implications for future research

Nivison et al.'s (2026) analyses also expose several key gaps in our understanding of the consequences of sensitive caregiving. For example, the majority of research on this topic has focused on children's attachment security and behavior problems. Less than a quarter of the studies included in this umbrella meta‐analysis examined children's cognition and language skills, even though these outcomes are as tightly tethered to experiences of caregiver sensitivity as the socioemotional ones. There also is a striking absence of meta‐analyses focused on the associations between sensitive caregiving and children's social competence with peers. This theoretically relevant outcome has been examined in individual studies, but these findings have not yet been examined meta‐analytically. Further meta‐analytic research on children's social competence as well as components of children's self‐regulation will provide valuable additional pixels in the emerging picture of the consequences of caregiver sensitivity for children's psychological and behavioral development.

It is equally important to consider novel domains of functioning that may be shaped by children's caregiving experiences. Physical health is one such domain. Health psychology theories emphasize that cardiovascular, metabolic, and immune system functioning across the lifespan are rooted in childhood experiences with caregivers (e.g., Uchino, 2009), and this has been supported by growing evidence from prospective longitudinal studies (Farrell et al., 2019). Continuing to expand the scope of inquiry to examine physical health outcomes alongside psychological and behavioral ones is likely to prove fruitful for sensitive caregiving research.

As we refine and expand our understanding of the domains of child development that may be impacted by sensitive caregiving, it is equally important to carefully consider how sensitivity is conceptualized and measured. One important distinction is the specific type of signals caregivers are responding to, namely, infant distress versus non‐distress cues. Most studies are not well positioned to evaluate whether caregivers' responses to these different signals have unique consequences for children's development because the caregiver–child interaction tasks typically focus on play or caretaking. Although these types of interactions afford valuable insights into how caregivers support children's exploration and respond to their verbal and nonverbal communications, they provide limited information on how caregivers respond to children's expressions of sadness, anger, or anxiety. Capturing those caregiver behaviors will require developing protocols for systematically eliciting these aspects of child distress in ecologically valid ways, both in individuals' homes and in the lab (e.g., Lee, Kuzava, & Bernard, 2022). Nivison et al. (2026) also highlight the potential value of considering caregiving‐related dimensions other than sensitivity. Their systematic review revealed that several other constructs—such as caregivers' expressions of warmth and negativity and their exercise of control—are associated with children's outcomes and the strength of those associations is comparable in size to those involving sensitive caregiving. These findings do not necessarily undermine the role of caregiver sensitivity in children's development. Rather, they point to the multifaceted roles that caregivers play in their children's lives (Sroufe, Egeland, Carlson, & Collins, 2005). Meaningful progress in this area will require thoughtful consideration of how different dimensions of caregiving work together to shape different aspects of children's development. At the same time, factor analytic work will provide valuable information about whether measures of theoretically distinct constructs reflect different or common phenomena.

A persistent critique of sensitive caregiving research is the overreliance on samples of mothers who are relatively affluent and living in Western countries. Nivison et al. (2026) demonstrate that scholars have responded to these concerns by involving fathers, parents from low or mixed socioeconomic backgrounds, and families from at least 35 countries. At the same time, there is room for continued improvement in the representativeness of the samples that participate in this research. Recruiting samples with diverse characteristics will provide much‐needed opportunities to empirically resolve long‐standing debates about the universality versus culturally specific consequences of children's caregiving experiences.

## Practical implications

Because Nivison et al. (2026) synthesized studies that examined the *associations* between caregiving experiences and children's outcomes, caution is warranted when drawing causal inferences based on these results. Randomized controlled trials of parenting‐based interventions provide especially powerful tests of the causal effects of caregiving experiences on children's development. That said, Nivison et al.'s (2026) findings do offer guidance for research evaluating the efficacy or effectiveness of caregiving‐focused interventions. First, they lend support to the idea of targeting sensitive caregiving behaviors and suggest that these types of interventions may have beneficial effects across multiple domains of development. Second, they highlight the importance of considering child language and cognitive functioning as intervention outcomes. Indeed, randomized controlled trials that only examine children's behavior problems may underestimate the full benefits of sensitivity‐focused interventions. Third, they indicate that interventions aiming to promote caregiver sensitivity may be especially impactful among socioeconomically disadvantaged families.

Existing evidence from parenting‐based interventions offers support for each of these ideas (Jeong, Franchett, Ramos de Oliveira, Rehmani, & Yousafzai, 2021). Specifically, early parenting interventions lead to meaningful improvements in multiple child development outcomes. The beneficial effects on children's cognitive and language skills are comparable to children's attachment security and socioemotional functioning, and the effects tend to be larger in low‐ and middle‐income countries than in high‐income countries. A critical but unresolved question concerns the durability of these intervention effects over the life course.

## Conclusion

The paper by Nivison and colleagues (2026) represents a valuable synthesis of five decades of research on the significance of sensitive caregiving for children's development. Their umbrella meta‐analysis summarizes the results of hundreds of studies involving tens of thousands of participants. The findings indicate that this extensive body of work was not vain and provide compelling support for the idea that caregiver sensitivity is a key determinant of healthy child development. At the same time, the findings also underscore that this work is not yet complete and offer a roadmap for future research clarifying the full scope of the impacts of these and other caregiving experiences for children's development.

## Ethical considerations

Ethical approval is not applicable to this commentary article.

## Data Availability

Data sharing is not applicable to this article as no datasets were generated or analyzed during the current study.
